# The Mnemonic Effects of Novelty and Appropriateness in Creative Chunk Decomposition Tasks

**DOI:** 10.3389/fpsyg.2018.00673

**Published:** 2018-05-09

**Authors:** Xiaofei Wu, Yu Liu, Jing Luo

**Affiliations:** Beijing Key Laboratory of Learning and Cognition, The Collaborative Innovation Center for Capital Education Development, School of Psychology, Capital Normal University, Beijing, China

**Keywords:** novelty, appropriateness, insight, Zeigarnik-like effect, creative, chunk decomposition tasks

## Abstract

Creativeness has been widely recognized as the ability to generate thoughts that are both novel (new) and appropriate (useful) (Barron, 1955). In this paper, we investigated the mnemonic effects of novelty and appropriateness in chunk decomposition tasks. Studies 1 and 2 utilized classical recognition tasks (explicit memory) and ambiguous word identification tasks (implicit memory) to reveal whether novelty and appropriateness are involved in different mnemonic systems. A 2 (familiarity) × 2 (appropriateness) experimental design was utilized in our experiments, and the four conditions were familiar-appropriate, familiar-inappropriate, novel-appropriate and novel-inappropriate. The results indicated that insight induced by novelty (novel-appropriate condition) has a better performance than other conditions; and further, found an interesting phenomenon of Zeigarnik-like effect which referred to remembering uncompleted tasks better than completed tasks (Zeigarnik, 1927). We further conducted Study 3 to ask participants to recall the encoding process (how the characters had been decomposed in the learning stage), which was more sensitive to Zeigarnik effect and indicated that performance of familiar-appropriate condition (uncompleted tasks) was better than other conditions.

## Introduction

Creativeness has been widely recognized as the ability to generate thoughts that are both novel (original, new, and unexpected) and appropriate (useful, valuable, and adaptive) (Barron, 1955). Insight, as a crucial mental foundation and a main form of creative thinking, has always been a research topic of interest to psychologists. It often requires that the problem solver remove the unnecessary constraint he/she has inappropriately imposed on the problem and become aware of a novel concept that is suitable to represent and to solve the problem. Insight research could provide further understanding of the creative nature and underlying mechanisms (Ohlsson, 1984a,b, 1992; Luo et al., 2004).

It is often assumed that insight experiences facilitate memory effects; this point has been implicitly demonstrated not only by utilizing human subjects with the nine-dots problem, in which subjects are asked to connect nine dots (3 × 3) by drawing straight and continuous lines that pass through each of the dots without lifting the pen from the paper (Scheerer, 1963), and other insight problems (Dominowski and Buyer, 2000; Wills et al., 2000) but also by utilizing animal (ape) subjects (Kohler, 1921). Pamela et al. (1979), however, first designed an elaborate experiment to prove the facilitation effects of insight on later memory. In their study, some incomprehensible sentences were presented to participants (for example, “The haystack is important because the cloth ripped”), and after 5 s, a cue (parachute) was provided to reveal the meaning of the sentence and help them understand what was previously confusing. Generally, participants would have an “aha” reaction when they received the cue, and the results suggested that there was a facilitating effect on the recall of sentences with the “aha” reaction compared with that of sentences with no “aha” reaction. In accordance with this result, Danek et al. (2013), utilizing magic tricks to induce the “aha” experience, found that self-generated insight could also facilitate recall performance. In their research, participants were asked to determine how the present trick was achieved, and upon discovering the solution, to report whether they had experienced insight during the solving process. In a recall task 2 weeks later, the recall performance of the previously reported insight solution was significantly greater than that of the no-insight conditions.

The above studies mainly investigated the insight memory effect of creative processes, but no studies have examined the memory effect of creativeness, especially the separation of novelty and appropriateness. As a result, previous creativeness studies mainly focused on the novelty feature of creativeness, suggesting that novelty could induce insight experience, while ignoring the appropriateness feature of creativeness. However, creativeness consists of both novelty and appropriateness, and it is possible that appropriateness is as important as novelty. Our present study was designed, first, to repeat the results of the facilitatory effects of insight induced by novelty, but, more importantly, to investigate the memory effects of appropriateness. Chinese character chunk decomposition (CD) tasks are an excellent way to separate the novelty and appropriateness of creative thinking. CD refers to the decomposition of familiar patterns into their component elements. This process is automatic and is followed by regrouping in another meaningful manner. Such regrouping is necessary, and as a result, during the problem solving process, the problem elements become automatically decomposed into familiar patterns. These automatic grouping processes may prevent us from forming appropriate and new mental representations that are critical for successful insight problem solving (Huang et al., 2015; Wu et al., 2017). Utilizing the matchstick arithmetic task, Knoblich et al. (1999, 2001) performed pioneering work in CD tasks. For example, to transform a false arithmetic statement “VI = VII + I” into a true equation, the numeral “VII” should be decomposed to “VI” and “I,” and the decomposed “I” is then moved to the left of the equal sign to form the true equation “VII = VI + I.” This example is a familiar decomposing pattern, and the decomposing process is automatic and easy. However, to transform “XI = III + III” to “VI = III + III,” the process of decomposing the “X” to “∖” and “/” is required and then regroups to “V.” This process is not automatic. Follow-up studies on CD using Chinese characters as materials were developed by Luo et al. (2006), Tang et al. (2009, 2016), Wu et al. (2009, 2010, 2013, 2017), Huang et al. (2015). Chinese characters are ideal examples of perceptual chunks that are composed of radicals, which in turn are composed of strokes. Strokes are the most simple and basic components of a Chinese character, and isolated strokes usually do not carry meaning. In contrast, radicals convey information about the meaning and pronunciation of the character. According to the theory of CD (Knoblich et al., 1999), it is much easier to separate a character into its radicals than to separate a character into its strokes because particular strokes are tightly embedded within a given perceptual chunk. In other words, the decomposition of characters into strokes requires a specific creative insight process that breaks the tight bond among the strokes created by the perceptual chunk. These properties enabled us to manipulate the novelty of CD: radical-level CD represents familiar processing, while stroke-level CD is novel processing. Moreover, the two manners of decomposition (familiar and novel) could generate either a true (appropriate) or a false (inappropriate) character, which enabled manipulation of the appropriateness of CD (Huang et al., 2015). Thus, novelty and appropriateness were manipulated in each CD task to generate four conditions: Novel-Appropriate, Novel-Inappropriate, Familiar-Appropriate, and Familiar-Inappropriate.

In this paper, we investigate the mnemonic effects of the novelty and appropriateness of creative CD tasks. The experiments included three parts: the learning stage, delay stage and testing stage. At the learning stage, the different manners of decomposition were presented to participants, and the participants were requested to state whether this manner could generate an appropriate character. We manipulated the novelty by exposing the radical or stroke-level decomposition and manipulated the appropriateness according to whether a true or false character could be generated by removing the required radical or stroke. Following the simple delay task, participants were asked to perform mnemonic tasks. We assumed that the novel condition, especially the Novel-Appropriate, which possibly requires a creative insight process (“aha” experience), would generate a better performance than the other conditions (Pamela et al., 1979; Dominowski and Buyer, 2000; Wills et al., 2000). Importantly, we further aimed to identify the different memory effects of novelty and appropriateness. Thus, studies 1 and 2 investigated memory effects using classical recognition tasks (explicit memory) and ambiguous word identification tasks (implicit memory). Because both studies 1 and 2 found an interesting phenomenon known as the Zeigarnik-like effect, which refers to remembering uncompleted or uncompleted tasks better than completed tasks (Zeigarnik, 1927), we added Study 3 to investigate the memory effect of the encoding process (by asking participants to recall how to decompose the character in the learning stage), which was more sensitive to the Zeigarnik-like effect. We expected to reveal whether the novelty and appropriateness of creative thinking are involved in different mnemonic systems by comparing the participants’ behavioral reactions.

## Study 1: the Mnemonic Effects of Novelty and Appropriateness in Explicit Memory Tasks

Explicit memory refers to tasks in which people are required to directly evaluate their memory. Generally, researchers have used the classical memory task – a recognition test in which respondents judge whether the present object appeared previously – to measure memory performance. In experiment 1, we investigated whether there would be a different memorization effect of novelty and appropriateness by utilizing separate recognition tasks.

### Participants

Thirty-seven undergraduate or graduate students recruited from Capital Normal University and Beijing Forestry University, and with a mean age of 23.2 years (*SD* = 2.9, range = 20–26) were paid to take part in this study. All of the participants were very familiar with Chinese characters and had normal or corrected-to-normal vision, were right-handed, and reported no history of neurological or psychiatric illnesses. Before the experiment, they all signed the informed consent forms approved by the Capital Normal University’s Committee on Activities Involving Human Subjects. The data of two participants were excluded from the final analysis because their performance was lower than 3 standard deviations of the mean; therefore, the final sample consisted of 35 participants (18 females).

### Method

#### Materials

**FIGURE 1:**
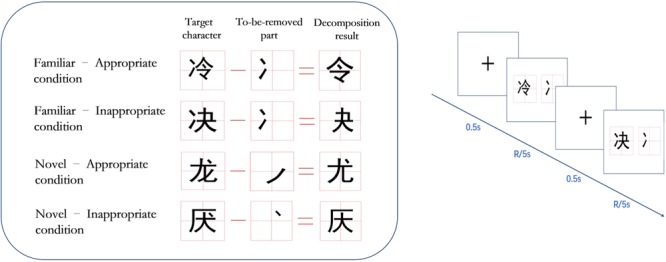
The left side shows examples of the materials used in the four conditions. (1) Familiar-appropriate condition: the Chinese character 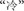 (leng, meaning cold) can be decomposed into 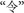 (ling, meaning to order) by removing the radical 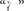; (2) familiar-inappropriate condition: when the radical 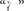 is removed from the character 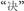 (jue, meaning resolve), the result is 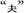 (false character); (3) novel-appropriate condition: the character 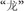 (long, meaning dragon) can be decomposed into 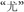 (you, meaning special) by removing the stroke 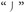; (4) novel-inappropriate condition: the character 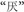 (yan, meaning disgust) can be decomposed into 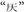 (false character) by removing the stroke 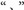. The right side of the figure shows the procedure of the learning stage.

A 2 (familiarity) × 2 (appropriateness) experimental design was utilized in this study. The four conditions were familiar-appropriate, familiar-inappropriate, novel-appropriate, and novel-inappropriate (see the left side of **Figure 1**). The materials of the familiar-appropriate condition consisted of characters that could be decomposed into a real character by removing the radical. For example, the Chinese character 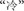 (leng, meaning cold) can be decomposed into 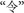 (ling, meaning to order) by removing the radical 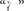. The materials of the familiar-inappropriate condition consisted of characters that could be decomposed into a non-real character by removing a radical. For example, after removing the radical 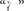 of the character 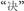 (jue, meaning resolve), the result is 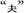 (a false character). The materials of the novel-appropriate condition consisted of characters that can be decomposed into a real character by removing a stroke. For example, the character 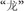 (long, meaning dragon) can be decomposed into 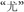 (you, meaning special) by removing the stroke 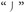. The materials of the novel-inappropriate condition consisted of characters that can be decomposed into a false character by removing a given stroke. For example, the character 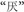 (yan, meaning disgust) can be decomposed into 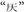 (a false character) by removing the stroke 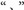. Each decomposition task consisted of two parts: the target character (the character to be decomposed) on the left and the part to be removed (a given stroke or radical) on the right. The learning stage materials consisted of 160 Chinese characters (old characters), with 40 characters for each condition. An additional 160 characters (new characters), matched to the old characters in terms of the familiarity and number of strokes, were used to investigate the recall performance of old characters in the testing stage. The familiarity of the Chinese characters was rated by three Chinese graduate students, and neural familiarity (*M* = 4.8, 7-point scale) and similar stroke number (*M* = 7.61) were selected as the formal materials measurements. Thus, the materials of this experiment consisted of 320 Chinese characters, half of which (160, 40 for each condition) were utilized in the task learning stage and all of which (320) were utilized in the testing stage.

#### Procedure

There were three stages of the experiment: the learning stage, delay stage, and testing stage. In the decomposing-task stage, the participants were exposed simultaneously to the character to be decomposed (target character) and the part to be moved (stroke or radical in the corresponding location) for 5 s and were required to judge whether the decomposition was appropriate by pressing a response key as soon as possible. After they pressed the key or after 5 s had passed with no response, the next trial was presented, and they were required to solve the new task immediately (see the right side of **Figure 1**). The 160 trials were divided into two blocks, and the sequence of blocks was balanced across all participants. For each block, the presentation order of the trials was randomized. After the learning task, the participants were asked to immediately perform a task where the participant continuously subtracted 3 from a random number (such as 204) for 1 min, which served as a delay task between the learning task and testing task. This delay task was chosen because it allowed the participants a short rest (due to the ease of the delay task for Chinese) while simultaneously preventing them from further processing the learning task before the testing task. After 1 min, the participants were asked to stop the mathematical computation task immediately and start the testing task. In the testing stage, participants were randomly exposed to 320 characters (half of which had been presented in the learning stage) one by one and were required to press the corresponding key to judge whether the character had been seen in the learning stage. The accuracy (ACC) and reaction time (RT) were recorded automatically by the E-Prime program.

### Results and Discussion

**FIGURE 2:**
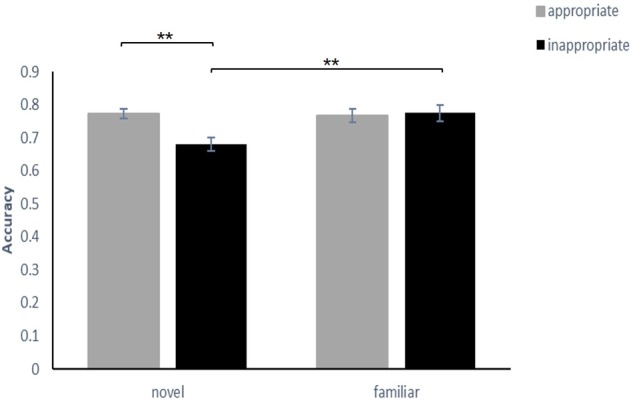
Comparisons of the accuracy of the four conditions in experiment 1. The error bars (capper vertical bars) represent the standard error. ^∗∗^Indicates a significant difference at *p* < 0.01.

A 2 (familiarity: familiar, novel) × 2 (appropriateness: appropriate, inappropriate) repeated-measures ANOVA was conducted to examine the ACC and RT of the participants’ correct recognition. The ACC was computed as the proportion of hits (the number of correctly recognized old characters/40) minus the average proportion of false alarms (the total number new characters judged as old characters/160) in each condition. RT reflected the mean RT of trial hits in each condition. For ACC, the results indicated that the main effect of familiarity between the familiar condition and the novel condition [*F*_(1,34)_ = 10.49, *p* < 0.01, $\eta_{p}^{2}$ = 0.24] was significant, and the main effect of appropriateness between the appropriate condition and the inappropriate condition was significant [*F*_(1,34)_ = 7.98, *p* < 0.01, $\eta_{p}^{2}$ = 0.19]. The interaction effect was significant [*F*_(1,34)_ = 11.75, *p* < 0.01, $\eta_{p}^{2}$ = 0.26]. A simple effect analysis showed that the ACC in the appropriate condition (*M* = 77.36%, *SEM* = 1.5%) was significantly greater than that in the inappropriate condition (*M* = 68.07%, *SEM* = 2.1%) at the novel level (*p* < 0.01) but showed no significant differences between the appropriate condition (*M* = 76.79%, *SEM* = 2.0%) and the inappropriate condition (*M* = 77.43%, *SEM* = 2.4%) at the familiar level (*p* = 0.11). Moreover, the ACC of the familiar condition was significantly greater than that of the novel condition at the inappropriate level (*p* < 0.01), but there was no difference between the familiar condition and the novel condition at the appropriate level (*p* = 0.10) (**Figure 2** and **Table 1**).

**Table 1:** Reaction time and accuracy of the four conditions in experiment 1, with standard error in parentheses.

	Novel-Appropriate	Novel-Inappropriate	Familiar-Appropriate	Familiar-Inappropriate
ACC	77.36% (1.5%)	68.07% (2.1%)	76.79% (2.0%)	77.43% (2.4%)
RT	1257 ms (62 ms)	1345 ms (64 ms)	1237 ms (62 ms)	1200 ms (59 ms)

**FIGURE 3:**
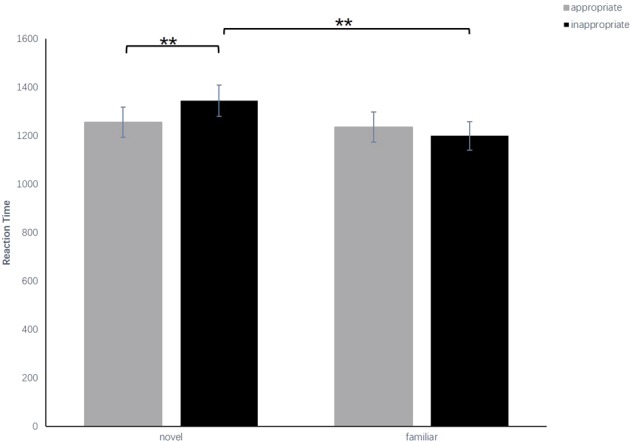
Comparisons of the reaction time for the four conditions in experiment 1. The error bars (capper vertical bars) represent the standard error. ^∗∗^Indicates a significant difference at *p* < 0.01.

For RT, the results showed that the main effect of familiarity between the familiar condition and the novel condition [*F*_(1,34)_ = 17.30, *p* < 0.001, $\eta_{p}^{2}$ = 0.37] was significant, and the main effect of appropriateness between the appropriate condition and the inappropriate condition was marginally significant [*F*_(1,34)_ = 4.02, *p* = 0.053, $\eta_{p}^{2}$ = 0.11]. The interaction effect was significant [*F*_(1,34)_ = 11.35, *p* < 0.01, $\eta_{p}^{2}$ = 0.26]. A simple effect analysis showed that the RT in the appropriate condition (*M* = 1257 ms, *SEM* = 62 ms) was significantly shorter than that in the inappropriate condition (*M* = 1345 ms, *SEM* = 64 ms) at the novel level (*p* < 0.01) but showed no significant differences between the appropriate condition (*M* = 1237 ms, *SEM* = 62 ms) and the inappropriate condition (*M* = 1200 ms, *SEM* = 59 ms) at the familiar level (*p* = 0.23). Moreover, the RT of the familiar condition was significantly slower than that of the novel condition at the inappropriate level (*p* < 0.01), but there was no significant difference between the familiar condition and the novel condition at the appropriate level (*p* = 0.13). The results for RT were consistent with those for ACC (**Figure 3** and **Table 1**).

Study 1 employed a classical learning-testing paradigm to uncover how novelty and appropriateness influence people’s explicit memory. Chinese characters were selected as the materials of creative CD tasks, as they could effectively be used for four conditions: novel-appropriate, novel-inappropriate, familiar-appropriate and familiar-inappropriate. Since our main aim was to reveal the mnemonic effects of creative thinking, we focused on comparing the novel-appropriate condition to other conditions. The results showed that the performances of the novel-appropriate condition were better than those of the novel-inappropriate condition, thus revealing the effect of appropriateness. We supposed that valid and successful decomposition perhaps gave the participants positive feedback and that the positive emotion (compared with neutral emotions) facilitated the memory effect (Kensinger, 2004; Kensinger and Corkin, 2004). Further, there was no difference between the novel-appropriate condition and the familiar-appropriate condition, revealing that the insight effects of novelty disappeared and that the positive emotion may have induced success, especially the success of creativeness. Other studies have found that the memory of insight accompanied by an “aha” experience performs better than that without an “aha” experience (Pamela et al., 1979; Wills et al., 2000; Danek et al., 2013). These findings were not in accordance with our research, which found no facilitating effect of novel-appropriate in comparison to familiar-appropriate. We speculated that there may be a reward of success in both our study and other insight studies that could facilitate memory performance. To investigate whether the reward of success or the insight of novelty results in better memory effects, we added an implicit memory task – ambiguous character recognition – in Study 2.

## Study 2: the Effects of Novelty and Appropriateness in Implicit Memory Tasks

In contrast to an explicit memory test, an implicit memory test can unconsciously affect thoughts and behaviors (Schacter, 1987). In Study 2, the same materials used in Study 1 were utilized in the learning stage; however, in the testing stage, participants were asked to undergo an implicit memory test that required them to judge whether a presented fuzzy character was a true character. We hypothesized that the mnemonic effects of novelty insight would be found in the implicit tasks.

### Participants

Thirty-three college students (15 females; *M* = 22.2 years, *SD* = 1.8, range = 21–25) who were recruited from Capital Normal University and who had not participated in experiment 1 were paid to take part in this study. All of the participants were very familiar with Chinese characters and had normal or corrected-to-normal vision, were right-handed, and reported no history of neurological or psychiatric illness. Before the experiment, they all signed the informed consent forms approved by the Capital Normal University’s Committee on Activities Involving Human Subjects.

### Method

#### Materials

The materials of the learning stage were consistent with those used in experiment 1. However, the fuzzy characters were selected at the testing stage. Using the Photoshop CS6 field blur treatment to weight the average of each pixel to control its degree of fuzziness, we selected a fuzziness of 75 pixels to create the fuzzy Chinese characters. Then, we invited ten people to determine whether they could recognize the current fuzzy character as a real character; when their average ACC was higher than 80%, we addressed the ambiguity again until the subjects’ ACC was less than 80%. Even though our study wanted to investigate the implicit memory effect, there were too many similar Chinese characters and if giving a higher ambiguous level, they would be make more mistakes. This was not our aim and thus, we chose a lower ambiguous level. All 320 materials, including 160 old characters, 80 new characters and 80 non-true characters, were processed to be fuzzy with the Photoshop software.

#### Procedure

**FIGURE 4:**
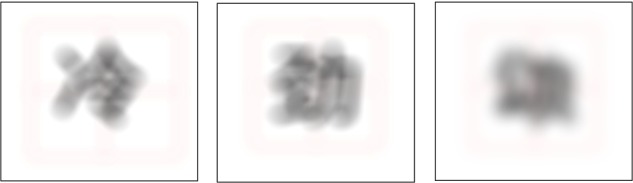
Examples of the materials used in the testing stage. **Left**: old character; the example is the character 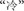 which was presented in the learning stage. **Middle**: new character; the example is the character 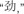 which was not presented in the learning stage. **Right**: false character; the example is the false character 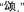

There were three stages in experiment 2: the character learning stage, delay stage and testing stage. In the learning stage (similar to experiment 1), the participants were exposed simultaneously to the character to be decomposed (target character) and the part to be moved (stroke or radical in the corresponding location) for 5 s and were required to judge whether the decomposition was appropriate by pressing a response key as soon as possible. After they pressed the key or after 5 s had passed with no response, the next trial was presented, and they were required to solve the new task immediately. The 160 trials were divided into two blocks, and the sequence of blocks was balanced across all participants. For each block, the presentation order of the trials was randomized. After the learning task, the participants were asked to immediately perform a task where the participant continuously subtracted 3 from a random number (such as 204) for 1 min, which served as a delay task between the learning task and testing task. After 1 min, the participants were asked to stop the mathematical computation task immediately and start the testing task. In the testing stage, the participants were exposed to 320 fuzzy characters: 160 of the characters had been presented (with non-fuzzy presentation) in the learning stage (old characters), 80 characters had not been seen in the learning stage (new characters) and 80 were false characters (**Figure 4**). The participants were required to judge whether the character presented was a true character (true–false character decision task) by pressing the corresponding key. The ACC and RT were recorded by the E-Prime software.

### Results and Discussion

**FIGURE 5:**
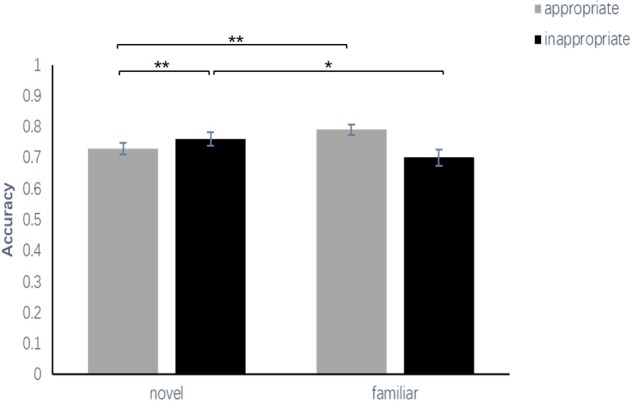
Comparisons of the accuracy of the four conditions in experiment 2. The error bars (capper vertical bars) represent the standard error. ^∗^Indicates a significant difference at *p* < 0.05; ^∗∗^ indicates a significant difference at *p* < 0.01.

A 2 (familiarity: familiar, novel) × 2 (appropriateness: appropriate, inappropriate) repeated-measures ANOVA was conducted to examine the ACC of the participants’ solutions. The ACC was computed as the proportion of hits (the number of correctly identified true characters/40) minus the average proportion of false alarms (the number of non-true characters identified as true characters/80) in each condition. The results showed that the main effect of appropriateness between the appropriate condition and the inappropriate condition [*F*_(1,32)_ = 25.84, *p* < 0.05, $\eta_{p}^{2}$ = 0.15] was significant, the main effect of familiarity between the familiar condition and the novel condition was not significant and the interaction effect of familiarity and appropriateness was significant [*F*_(1,32)_ = 30.41, *p* < 0.001, $\eta_{p}^{2}$ = 0.49]. A simple effect analysis showed that the ACC in the novel condition (*M* = 79.32%, *SEM* = 1.8%) was significantly greater than that in the familiar condition (*M* = 72.96%, *SEM* = 1.9%) at the appropriate level (*p* < 0.01) and that the ACC in the familiar condition (*M* = 75.76%, *SEM* = 2.2%) was significantly greater than that in the novel condition (*M* = 70.23%, *SEM* = 2.7%) at the inappropriate level (*p* < 0.01). Meanwhile, a simple effect analysis showed that the ACC in the appropriate condition (*M* = 79.32%, *SEM* = 1.8%) was significantly greater than that in the inappropriate condition (*M* = 70.23%, *SEM* = 2.7%) at the novel level (*p* < 0.01), and there was no significant different between the appropriate condition and the inappropriate condition at the familiar level (*p* = 0.38) (**Figure 5** and **Table 2**).

**Table 2:** Reaction time and accuracy of the four conditions in experiment 2, with standard error in parentheses.

	Novel-Appropriate	Novel-Inappropriate	Familiar-Appropriate	Familiar-Inappropriate
ACC	79.32% (1.8%)	70.23% (2.7%)	72.96% (1.9%)	75.76% (2.2%)
RT	596 ms (20 ms)	600 ms (20 ms)	594 ms (20 ms)	602 ms (20 ms)

For RT, there was no significant difference between the main effects and interactive effect (**Table 2**).

The results of Study 2 proved the mnemonic effects of novelty insight, meaning the memory performances of the novel-appropriate condition was significantly better than both that of the novel-inappropriate condition and that of the familiar-appropriate condition. This study, utilizing the high-validity implicit memory test, supported our hypothesis that novelty, which possibly requires a creative insight process, induced a better memory effect. This finding was consistent with previous studies that found the memorization of insight experience (Pamela et al., 1979; Wills et al., 2000; Danek et al., 2013).

In both Studies 1 and 2, there was an interesting finding that the memorization effect of the familiar-inappropriate condition was greater than that of the novel-inappropriate condition. We argued that this phenomenon was the separation of the Zeigarnik-like effect in a familiar (easy) condition and a novel (difficult) condition. The Zeigarnik effect refers to a person remembering uncompleted or interrupted tasks better than completed tasks (Zeigarnik, 1927). Similarly, people may remember non-real situations better than real (exact) situations, especially in a familiar situation. Perhaps people are not able to tolerate an easier non-real result (familiar-inappropriate condition) than a relatively difficult non-real result (novel-inappropriate). If the Zeigarnik-like effect in fact existed in our studies, we expected the memorization performance to be better in the familiar-inappropriate condition than in the familiar-appropriate condition, which included the pure Zeigarnik-like effect, thus revealing the effect of inappropriateness. We know that the Zeigarnik effect was more sensitive in the encoding stage, and when the process of problem encoding or solving is interrupted, the problem situation is retained in the mind, thus leading to a better memory performance (Zeigarnik, 1927). Based on that concept, we designed experiment 3, which required participants to remember the encoding (decomposition) process, and hypothesized that we would find the obvious Zeigarnik-like effect by asking participants to remember the encoding process specifically, meaning that the memorization performance would be better in the familiar-inappropriate condition than in the novel-inappropriate condition and the familiar-appropriate condition.

## Study 3: the Memory Effects of Novelty and Appropriateness in the Encoding Process

Based on the findings of Studies 1 and 2, in Study 3, we further examined whether there was a Zeigarnik-like effect in CD tasks by asking participants to recall how the exposed character had been decomposed in the learning stage (meaning the task encoding stage). It is argued that to obtain the Zeigarnik effect, it is better to interrupt the problem when the participant is “most engrossed” (Zeigarnik, 1927). We hypothesized that performance would be better in the inappropriate condition than in the other conditions, meaning there would be a Zeigarnik-like effect; moreover, we hypothesized that performance would be better in the familiar-inappropriate condition than in the novel-inappropriate condition, meaning that the separation of the Zeigarnik-like effect in cases of an easier failure or incompleteness induced a better memory effect than in an uneasy situation, meaning mnemonic performance was better in the familiar-inappropriate condition than in the novel-inappropriate condition.

### Participants

Thirty college students (*M* = 22.9 years, *SD* = 1.6, range = 20–24), recruited from Capital Normal University, Beijing Forestry University, Beijing Institute of Technology and Beijing Science and Technology University and who had not participated in experiments 1 and 2, were paid to take part in this study. All of the participants were familiar with Chinese characters and possessed normal or corrected-to-normal vision, were right-handed, and reported no history of neurological or psychiatric illness. Before the experiment, they all signed the informed consent forms approved by the Capital Normal University’s Committee on Activities Involving Human Subjects. The data of three participants were excluded from the final analysis because their performance was lower than 3 standard deviations of the mean; therefore, the final sample consisted of 27 participants (15 females).

### Method

#### Materials

The materials were similar to those used in experiment 1.

#### Procedure

There were three stages in experiment 3: the learning stage, delay stage, and testing stage. In the learning stage, as in experiments 1 and 2, the participants were exposed simultaneously to the character to be decomposed (target character) and the part to be moved (stroke or radical in the corresponding location) for 5 s and were required to judge whether the decomposition was appropriate by pressing a response key as soon as possible. After they pressed the key or after 5 s had passed with no response, the next trial was presented, and they were required to solve the new task immediately. The 160 trials were divided into two blocks, and the sequence of blocks was balanced across all participants. For each block, the presentation order of the trials was randomized. After the decomposing task, the participants immediately performed the delay task, in which they were asked to continuously subtract 3 from a random number (such as 204) for 1 min. After 1 min, the participants were asked to stop the mathematical computation task immediately and start the testing task. The testing stage included two steps: in the first step, the participants were exposed to 320 characters (half of which had been presented in the decomposition stage) on a printed paper and asked to judge whether the character had been presented in the decomposing-task stage. If the answer was yes, then in the second step, they wrote down the removed part (stroke or radical) of the character to show how this character had been decomposed in the learning stage. If the answer was no, they went on to the next trial.

### Results and Discussion

**FIGURE 6:**
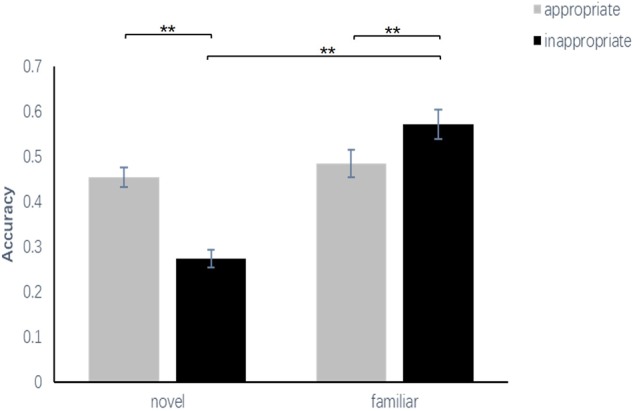
Comparisons of the accuracy of the four conditions in experiment 3. The error bars (capper vertical bars) represent the standard error. ^∗∗^indicates a significant difference at *p* < 0.01.

A 2 (familiarity: familiar, novel) × 2 (appropriateness: appropriate, inappropriate) repeated-measures ANOVA was conducted to examine the ACC of the participants’ solutions. The ACC was computed as the proportion of hits (the number of old characters that were correctly recalled how to decompose/40) minus the average proportion of false alarms (the total number new characters falsely recognized as old ones/160) in each condition. The results showed that the main effect of familiarity between the familiar condition and the novel condition [*F*_(1,26)_ = 57.70, *p* < 0.001, $\eta_{p}^{2}$ = 0.69] was significant, and the main effect of appropriateness between the appropriate condition and the inappropriate condition was significant [*F*_(1,26)_ = 11.57, *p* < 0.05, $\eta_{p}^{2}$ = 0.31]. The interaction effect was significant [*F*_(1,26)_ = 142.19, *p* < 0.001, $\eta_{p}^{2}$ = 0.85]. A simple effect analysis showed that the ACC in the appropriate condition (*M* = 45.37%, *SEM* = 2.2%) was significantly greater than that in the inappropriate condition (*M* = 27.41%, *SEM* = 2.0%) at the novel level (*p* < 0.01) and that the ACC in the inappropriate condition (*M* = 57.22%, *SEM* = 3.3%) was significantly greater than that in the appropriate condition (*M* = 48.42%, *SEM* = 3.0%) at the familiar level (*p* < 0.01). Moreover, the performances in the familiar-inappropriate condition were better than those in the novel-inappropriate condition (*p* < 0.01), but there was no difference between the novel-appropriate and the familiar-appropriate condition (*p* = 0.39) (**Figure 6**).

As the encoding process is more sensitive to the Zeigarnik effect, experiment 3 focused on examining the encoding (decomposing) stage. The results were consistent with our hypothesis that the memorization performance would be better in the familiar-inappropriate condition than in the novel-inappropriate condition and the familiar-appropriate condition. Meanwhile, we found that the performance in the familiar-inappropriate condition was better than that in the novel-inappropriate condition, meaning that the separation of the Zeigarnik-like effect in cases of an easier failure or incompleteness induced a better memory effect than in an uneasy situation.

## General Discussion

Utilizing the CD of Chinese characters as experimental materials, this study investigated the mnemonic effects of novelty and appropriateness. For CD tasks, radical-level CD represented familiar processing, while stroke-level CD represented novel processing. Moreover, the two manners of decomposition (familiar and novel) could generate either true (appropriate) or false (inappropriate) characters, which enabled manipulation of the appropriateness of CD (Huang et al., 2015). Thus, there were four conditions: novel-appropriate, novel-inappropriate, familiar-appropriate, and familiar-inappropriate. In the three different experimental tasks – recognition tasks, ambiguous word identification tasks and recalling the encoding process – we found that the mnemonic effect of novelty insight (novel-appropriate condition) and the separation of the Zeigarnik-like effect (i.e., performance was better in the familiar-inappropriate condition than in the novel-inappropriate condition) existed simultaneously in the CD tasks.

Many studies have found the mnemonic effect of insight, including induced perceptual insight (Ludmer et al., 2011), induced fuzzy sentence insight (Pamela et al., 1979) and self-generated insight from magic tricks (Danek et al., 2013). Our study adds to the literature by further indicating that inducing insight by novelty can facilitate subsequent memory performance. In contrast to the radical-level CD tasks, the task of stroke-level CD is novel and could induce an “aha” experience (the sudden realization of a new solution to the problem). All of our experiments, utilizing different memory tasks, revealed that the novelty insight of creative problem solving can facilitate mnemonic performance.

Our research found that mnemonic performance was better in the familiar-inappropriate condition than in the novel-inappropriate condition in all three experiments (and better than in the familiar-appropriate condition in the implicit memory experiment). Although not accordant with our hypothesis, we argued that a separation phenomenon existed for a Zeigarnik-like effect in a familiar (easy) condition and novel (difficult) condition, meaning that people remember non-real situations better than real (exact) situations, especially in familiar situations. In sum, the facilitating effect of novel-appropriate insight and the Zeigarnik-like effect of familiar-inappropriate insight were both found in CD tasks.

Our behavioral results were highly consistent with neural underpinning research by Huang et al. (2015). Similar to our study’s manner of decomposition, Huang et al.’s (2015) fMRI study utilized Chinese characters as materials and tried to separate the neural mechanisms of novelty and appropriateness. They found that the activation of the anterior cingulate cortex, hippocampus and amygdala was greater in the appropriate condition than in the inappropriate condition at the novel level. In our study, mnemonic performance was also better in this contrast, revealing that novelty insight facilitated mnemonic per-formance. Based on this result and Huang et al. (2015) work, we conjecture that the ACC could be responsible for specific conflict-detection during creative CD (Carter et al., 1998; Botvinick et al., 1999, 2001; Fan et al., 2007, 2008), the hippocampus to the formation of novel, task-related associations between the old nodes of knowledge (Luo and Niki, 2003; Zhao et al., 2013) and the amygdala to the emotional experience of the “aha!” moment (Paton et al., 2006; Sang and Hamann, 2007; Sass et al., 2014).

This study first systematically investigated the memory effect inducing by novelty and appropriateness utilizing the CD tasks. We found the facilitating effects of both novel insight and the Zeigarnik-like effect in familiar-failure to memory. Nevertheless, there were several limitations of this study. First, we hypothesized that novelty could induce an insight experience, thus further facilitating mnemonic performance, but we did not examine whether the participants experienced insight sense. Although previous research (Huang et al., 2015) has proved that the insight effect occurs in the novel-appropriate condition, insight sense should be measured to provide greater validity for the results. Second, combined with the research of Huang et al. (2015), we further analyzed the neural mechanisms of the memory process in CD tasks. Although the materials of the two studies are similar and the results are consistent, we did not directly investigate the neural underpinning of the mnemonic effect of novelty and appropriateness in our research. Thus, the brain mechanism underlying the mnemonic effect of novelty and appropriateness should be investigated in future studies.

## Author Contributions

YL and JL designed the experiment. JL supervised the research procedure. YL and XW collected and analyzed the data. XW and JL wrote the manuscript.

## Conflict of Interest Statement

The authors declare that the research was conducted in the absence of any commercial or financial relationships that could be construed as a potential conflict of interest.
